# Optimizing bacteriophage engineering through an accelerated evolution platform

**DOI:** 10.1038/s41598-020-70841-1

**Published:** 2020-08-19

**Authors:** Andrew H. Favor, Carlos D. Llanos, Matthew D. Youngblut, Jorge A. Bardales

**Affiliations:** 1 Nextbiotics Inc., Oakland, CA 94609 USA; 2 Present Address: Department of Materials Science and Engineering, University of California, Berkeley, CA 94720 USA

**Keywords:** Bacteriophages, Genetic engineering, Synthetic biology

## Abstract

The emergence of antibiotic resistance has raised serious concerns within scientific and medical communities, and has underlined the importance of developing new antimicrobial agents to combat such infections. Bacteriophages, naturally occurring bacterial viruses, have long been characterized as promising antibiotic alternatives. Although bacteriophages hold great promise as medical tools, clinical applications have been limited by certain characteristics of phage biology, with structural fragility under the high temperatures and acidic environments of therapeutic applications significantly limiting therapeutic effectiveness. This study presents and evaluates the efficacy of a new accelerated evolution platform, chemically accelerated viral evolution (CAVE), which provides an effective and robust method for the rapid enhancement of desired bacteriophage characteristics. Here, our initial use of this methodology demonstrates its ability to confer significant improvements in phage thermal stability. Analysis of the mutation patterns that arise through CAVE iterations elucidates the manner in which specific genetic modifications bring forth desired changes in functionality, thereby providing a roadmap for bacteriophage engineering.

## Introduction

Bacteriophages hold great potential as antimicrobial tools, which are increasingly needed within medical and industrial contexts. While the diversity and virulence of bacteriophages make them ideally suited for targeting a broad range of pathogenic bacteria, the efficacy of phage therapy has been significantly hindered by their inability to withstand the environmental conditions of such applications^1–6^. Much research has gone into the engineering of phages for the purpose of overcoming the challenges associated with their therapeutic use^7–10^. Most of these efforts have been rooted in hypothesis-driven approaches, which focus on enhancing phage activity through the addition of specific genetic circuits to enhance antimicrobial efficacy. While such methods can often lead to efficient optimization of a desired trait, they explore a limited space of possible solutions and therefore have a limited capacity to identify novel solutions^11,12^.

Directed evolution is a hypothesis-free approach that has been efficiently utilized in order to optimize the functionality of biomolecular systems through the improvement of selected traits^13,14^. At this time, however, limited research has utilized the iterative techniques of directed evolution for the optimization of bacteriophage stability and efficacy. Chemical mutagenesis with alkylating agents such as ethyl methanesulfonate (EMS) has long been characterized as an effective method for the induction of functionally relevant changes in bacteriophage genomes^15–17^, and has been used for the identification of mutations associated with desirable phenotypic changes in a variety of organisms^18–21^. Considering the pressing need for effective antibiotic alternatives, the positive receptibility of bacteriophages to chemical mutagenesis provides a powerful method for overcoming the limitations associated with the use of wild type bacteriophages as antimicrobial agents.

Here, we describe a directed evolution platform, CAVE, which utilizes the iterative coupling of mutagenesis to a desired selection criterion, in order to drive bacteriophage evolution towards an expressed phenotype conferring improved physical characteristics. In this study, we first use the *Escherichia coli* bacteriophage T3 as a model system to demonstrate the technological feasibility and efficacy of this approach. Over the course of 30 rounds of accelerated evolution, with each round being coupled to a thermal-selection filter, we gradually evolved a library of mutant variants of the bacteriophage T3 that possess dramatically improved thermal tolerance relative to the wild type virus. Following the development and physical characterization of the evolved mutants, genomic analysis of the mutant phage pools allowed us to map mutation patterns and their frequencies across different rounds of CAVE. Generation of phages with single mutations allowed us to identify the specific genes that lead to enhanced stability. This application was subsequently used to evolve the *Escherichia coli* phage T7, and two unique *Salmonella enterica* specific phages, NBSal001 and NBSal002. In all cases the evolved phages had a significant improvement in their thermal stability, demonstrating the efficacy and broad applicability of the CAVE methodology. Furthermore, analysis of the changes in protein sequence associated with these key mutations provides insight regarding which proteins and domains can be further engineered to induce desired phenotypic improvements. The sum of these results demonstrates an effective pipeline for the engineering of bacteriophages with enhanced stability, and provide an opportunity to improve a range of additional characteristics that can be used to overcome the most significant barriers that have traditionally limited the development of bacteriophage therapy.

**Figure 1:**
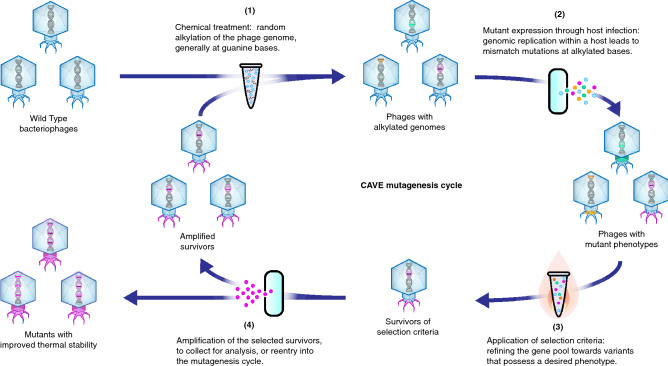
Schematic of the CAVE pipeline for bacteriophage directed evolution, with iterative cycles of mutagenesis and thermal selection. In this procedure, bases within the bacteriophage genomes are randomly affected by treatment with a chemical mutagen. Upon replication within a host, a mismatch mutation is introduced. The mutant library is then subjected to high-temperature incubation to select for thermally stable variants; surviving phages are amplified and carried over to subsequent iterations.

CAVE combines the introduction of random mutations across the phage genome with selective pressure to promote enrichment of the specific mutations that enhance a desired phenotypic property (Fig. 1). This directed evolution methodology follows several steps: (1) introduction of random mutations across the phage genome, (2) fixation of the random mutations through phage amplification in order to generate a pool of mutant phages, (3) selection of phage variants that possess the desired phenotype by application of selection criteria, and (4) amplification of the phage variants to collect for use, analysis, or reentry into the mutagenesis cycle. In our efforts to improve thermal stability, a progressively stringent selection condition of high-temperature incubation was used. Surviving phages were reintroduced to mutagenesis cycles until significant improvement in heat-resistance was observed.

**Figure 2:**
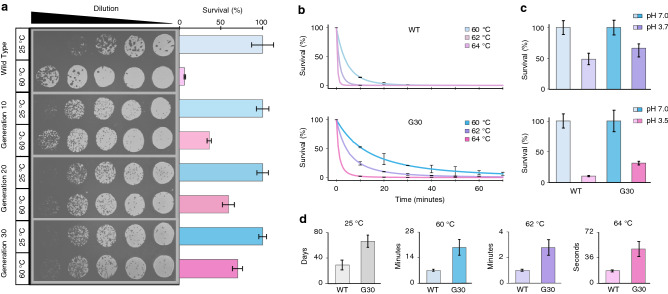
Improvement of thermal stability through directed evolution. (**a**) Multiple rounds of this procedure leads to increased resistance to thermal degradation. Incubation of T3 phages at 60 $^{\circ }$C for 1 h resulted in active phage survival of 6.6%, 36.0%, 59.1%, and 69.9% after 0, 10, 20, and 30 rounds of directed evolution, respectively. Relative concentrations were calculated using plaque-counting assays and linear regressions over four 40:140 $\upmu$L serial dilutions (n = 4); error bars representing ± SD. (**b**) The effect of temperature on degradation rate. The percent survival of wild type and mutant bacteriophages over the course of a 70 min incubation at 60, 62, and 64 $^{\circ }$C, quantified using a linear regression over serial dilutions (n = 3). Curves were fit to Eq. (1). Error bars represent the difference between theoretical curve values and measured titer for a given time point. (**c**) Mutant phages display improved tolerance to acidic conditions. Percent survival after 30 min incubation under acidic conditions, relative to neutral control conditions. (**d**) Half life values calculated for wild type and mutant phages at 25, 60, 62, and 64 $^{\circ }$C; error bars are ± SD. Relative concentrations were calculated using plaque-counting assays and linear regressions, with error bars representing ± SD. Theoretical curves were fit to experimental data using a fractional-order kinetic model and the Arrhenius law.

To determine the efficacy of CAVE at enhancing phage stability, we first subjected the T3 phage to 30 rounds of directed evolution. The thermal stability of these evolved phages was tested by quantifying the number of infective phage particles after incubation at a mild temperature (25 $^{\circ }$C) and at a high temperature (60 $^{\circ }$C). We observed that application of CAVE effectively improved T3’s ability to survive high temperature incubation, with percent survival increasing from 6.6 to 69.9% over the course of CAVE iterations (Fig. 2a). Interestingly, the improvement of phage survival occurs rapidly during initial rounds and slows down in later rounds, suggesting that the combined effect of multiple mutations on phage thermal stability ultimately reaches a plateau.

To have a more detailed understanding of the evolved phages’ thermal stability, time-dependent survival assays were performed for both wild type and 30th-generation evolved phages at high temperatures (Fig. 2b) as well as room temperature (Supplementary Figure S1). Time-dependent degradation data was used in a regression analysis, in order to fit the parameters of a kinetic model for bacteriophage thermal degradation^22^. We observed consistent and significant increases in the survival rates for the evolved phages when compared to the wild type T3 as a function of time. The time-dependent degradation data were used to calculate the half-lives of active wild type and evolved phages at each temperature tested. As expected, the evolved phages consistently had longer half-lives than the wild-type phages (Fig. 2d). Evolved phage stocks at mutagenesis generations 10, 20, and 30 are denoted “G10”,“G20”, and “G30”, respectively. Taken together, these results indicate a significant change in the kinetics of denaturation between the wild-type and evolved phages tested, with evolved phages displaying an improved ability to tolerate high temperatures for longer periods of time.

Importantly, CAVE applications lead to a broader enhancement of phage stability, as indicated by the evolved bacteriophages displaying enhanced tolerance to acidic conditions relative to their wild-type counterparts (Fig. 2c). The pH values of the acidic group were chosen based on their presence around the inflection point at which acidic degradation of these phages increases rapidly with small decreases in pH^23^.

Furthermore, to test if application of CAVE caused secondary effects beyond structural stabilization, we decided to test if unrelated traits associated with phage biology such as bacteriophage lytic activity or host range were affected. We observed that neither the infection dynamics (Supplementary Figure S2) nor the host range (Supplementary Figure S3) were changed in the evolved phages. These results demonstrate that the CAVE methodology can specifically and efficiently improve the stability of phages without inducing undesired changes in other functional traits.

**Figure 3:**
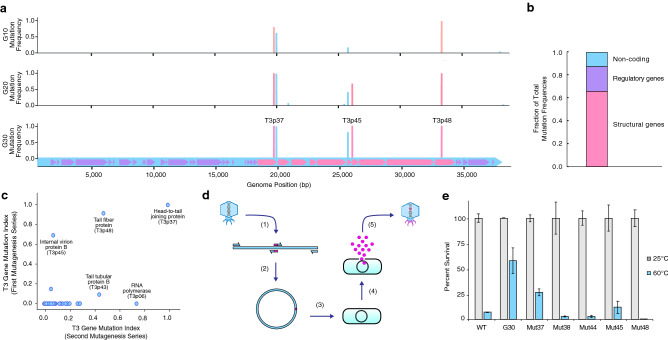
Genomic changes observed in the T3 bacteriophage genome over the course of 30 applications of the CAVE protocol. (**a**) Mutation frequencies observed in the T3 mutant gene pools after 10, 20, and 30 rounds of directed evolution. Mutation-frequency bars are color-coded corresponding to their functional genome location where they occur: blue are non-coding regions, magenta are structural genes, and purple are regulatory genes. (**b**) Fraction of net mutation frequencies observed within structural genes, regulatory genes, or non-coding regions, combined from final evolved generations of all T3 mutants from first and second CAVE series. (**c**) Comparison of mutation frequency indices at round 20 of CAVE application for two different series of T3 mutagenesis. (**d**) Protocol for recapitulation of mutations at specific sites using phage-rebooting. (**e**) Results from recapitulation of mutations at several structural genes verify that such mutations confer improved stability.

Following the completion of 30 rounds of CAVE application with the T3 phage, purified genomes from the T3 mutant pools at the 10th, 20th, and 30th generations of evolution were next-generation sequenced and analyzed through a variant-calling procedure to detect the presence and frequency of mutations relative to the wild type T3 reference genome (Fig. 3a). A limited number of mutations were observed across the phage genome during early rounds of mutagenesis, and such mutations become subsequently enriched, with many reaching saturation levels (Supplementary Figure S4). Importantly, the rate of mutation enrichment correlated with the enhancement of thermal stability seen in our high-temperature incubation assays (Supplementary Figure S5). The list of mutated genes and the locations of specific mutations can be found in Supplementary Table S1. Most of the observed mutations occured within coding sequences of structural genes, however, some mutations also occurred in non-coding regions (Fig 3b). The most prevalent mutations occurred within genes involved in structural assembly and the joining of subunits. Among the affected genes, the most commonly observed mutation regions appeared in genes such as the *head-to-tail joining protein* (T3p37), *tail-tubular proteins* (T3p42,43), and *internal virion proteins* (T3p44–47)—all genes which either encode protein subunits of the tail complex or are involved in the mechanical activities of structure-assembly and DNA injection. Most mutations in structural genes involved substitutions of amino acids that were larger and more hydrophobic than their wild-type counterparts (Supplementary Figure S6). Due to the prevalence of mutations at interfacial regions between protein subunits, we hypothesize that this trend arises from such mutations conferring improved hydrophobic packing between the components of the assembled tail complex. We also observed the elongation of a poly-guanine region as the directed evolution rounds progressed (Supplementary Figure S7), suggesting that regulatory sequences could also play an important role in enhancing the formation and activity of phage particles.

To understand the robustness of the CAVE method, we performed a second independent evolution series, starting again from wild type T3 phages, in order to determine if any characteristic mutation patterns would reappear this second time around, despite the random nature of CAVE. For the purpose of characterizing the tolerance of phages to chemical mutagenesis and to maximize the effectiveness of the CAVE protocol, the mutagen concentration in the second series was increased 180-fold relative to the initial application. This second application of CAVE did result in improvements in thermal stability that were similar to those observed in the first evolution series (Supplementary Figure S8), and also led to the generation of specific mutations and their enrichment across the evolved T3 phage genome (Supplementary Figure S9). Comparison of the total mutation frequencies from the first and second series of CAVE applications showed that specific genes were common mutation sites (Fig. 3c), most of which represent mutations in structural genes. Net-gene mutation frequencies were calculated as the sum of all mutation frequencies greater than 0.01 at each base position within a given gene’s coding region. This comparison demonstrated that two of the most mutagenized regions were the T3p37 and T3p45 genes. The high mutation rate at the T3p37 gene (coding for the *head-to-tail joining protein*, which bridges the capsid and tail of the bacteriophage^24^) further suggested that modification of this structure plays a critical role in conferring the improvement in structural stability observed over the course of directed evolution. The T3p45 gene codes for an *internal virion protein*, which assembles at the capsid interior, and participates in the DNA-injection process^25^. These results show that the primary sites of enriched mutations under a thermal selection criteria were genes involved in structural integrity, and that the mutations observed enhance the ability of these proteins to perform this function.

To validate the role of the observed mutations found in our genomic analyses, we decided to perform an in vitro assembly protocol followed by *phage rebooting*, as described in previous literature^7^, to generate a series of T3 phage variants, each with one of the single mutations found in the 30th-generation mutant gene pool, in order to assess their influence on survivability under high temperature conditions (Fig. 3d). We selected 5 mutations, 3 occurring in structural genes, including structural genes T3p37, T3p45, T3p48 (corresponding to synthetic mutants named Mut37, Mut45, and Mut48, respectively) and also at non-coding mutation sites (Mut44, Mut38; nomenclature based on their corresponding downstream gene). We observed that two mutations occurring within structural genes (T3p37 and T3p45) led to enhanced phage stability, supporting CAVE’s ability to enrich specific mutations involved in conferring improved thermal stability (Fig. 3e).

**Figure 4:**
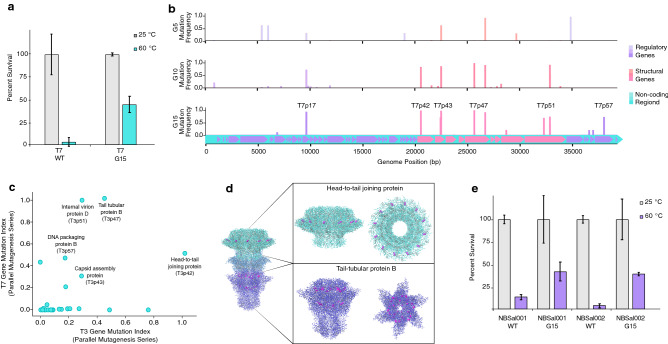
Application of CAVE protocol to other bacteriophage species. (**a**) Comparison of wild type vs. mutant T7 bacteriophage (after 15 rounds of directed evolution), percent survival following 1-h incubation at 25 $^{\circ }$C and 60 $^{\circ }$C. (**b**) Mutation frequencies observed in the mutant gene pools of the T7 bacteriophage after 5, 10, and 15 rounds of directed evolution. (**c**) Comparison of mutation frequency indices at round 20 of CAVE application between bacteriophages T3 and T7. (**d**) Crystal structures of the assembled T7 tail complex (PDB ID = 6R21), with high mutagenesis proteins: *head-to-tail joining protein*, *tail-tubular protein A*, *tail-tubular protein B* (mutation sites colored in magenta). (**e**) Salmonella phages NBSal001 and NBSal002: comparison of wild type vs. mutant (after 15 rounds of directed evolution), percent survival following 1-h incubation at 25 $^{\circ }$C and 60 $^{\circ }$C.

The T7 bacteriophage is a close relative of T3; the two phages belong to the *Podoviridae* family and have a similar genomic structure and share many of the same genes, which encode proteins of very similar amino acid sequences and functionality. Motivated by the extensive characterization of the T7 phage in previous literature, and the genetic similarity between these two species, we decided to evolve the T7 bacteriophage in parallel with the T3 phage, using the high-mutagen CAVE protocol described above. There were two primary goals of our parallel-evolution analysis: to determine if applying the same mutagenesis and selection conditions to two different species of bacteriophages would induce similar changes in thermostability, and to determine if mutations in common genes were correlated with changes in thermal stability. We observed that the CAVE protocol enhanced T7 phage stability (Fig. 4a) and that, in a similar manner to T3, a series of mutations enriched across the phage genome occur predominantly in structural genes (Fig. 4b). Mutations within several common genes were found to be enriched in both T3 and T7 phages over the course of directed evolution (Fig. 4c). The region coding for T7’s *head-to-tail joining protein* (T7p42 gene) was again a significant site of mutagenesis. Several other genes were prevalent sites of mutagenesis in both phages (Supplementary Table S1), including structural genes such as those encoding capsid assembly proteins, and internal virion proteins. The improvements seen in both species indicate that this approach is an effective way to induce phenotypic changes in different species of phages. Additionally, the convergent effects of mutagenesis seen in the genes shared by the T3 and T7 phages suggest that, despite the random nature of this protocol’s mutagenesis step, the steps involved in applying a given selection criterion guide the genomic changes within the experimental population towards semi-directional patterns of regional mutagenesis within structural genes. Analysis of the crystal structures of highly mutated proteins (Fig. 4d) indicate that many mutations occur at the interfacial regions between subunits of the assembled phage. We were able to map mutations sites (represented at magenta residues) from both species to the structures of three proteins (with corresponding gene-IDs): *head-to-tail joining protein* (T3p37, T7p42), *tail-tubular protein A* (T3p42, T7p46), and *tail-tubular protein B* (T3p43, T7p47), due to these proteins possessing a high degree of sequential and structural homology between the T3 and T7 phages.

Following initial results with *E. coli* phages T3 and T7, we hypothesized that the CAVE method can be broadly applicable to phages with different bacterial hosts. In order to test this, we decided to work with two Salmonella bacteriophages. The application of the CAVE technique with a high-temperature selection criteria was applied to these phages, using their host strain (*Salmonella enterica*) to foster their mutagenesis through lytic amplification. After 15 successive rounds of directed evolution with thermal selection, it was observed that the survivability of these phages at $60\,^{\circ }$C had increased by 30% (Fig. 4e). These results support the broad applicability of this technology to a diverse range of phage families with a multitude of target hosts.

The efficacy of this technique is dependent on the application of the chemical mutagenesis steps as well as the selection steps, as demonstrated by survival assays on control groups, where 20 rounds of directed evolution were performed on the T3 phage without the use of chemical mutagenesis or without the application of thermal selections (Supplementary Figure S10).

In consideration of how the introduction of various mutations might lead to the enhanced stability of our final product, we hypothesize that the most influential phenotypes for conferring tolerance to high temperatures are those that induce changes in protein structure and energetics. The effect of even single amino acid substitutions becomes amplified when expressed in multimeric structures, as is the case in of the 12-subunit head-to-tail connector proteins and tail tubular proteins, which were among the most affected genes in this study. The results of next-generation sequencing data analysis for the evolved phage populations indicate a trend towards genetic homogeneity rather than diversity. Indeed, despite the continuous application of chemical mutagenesis throughout this procedure, we hypothesize that the selective pressure of our thermal selection steps held more influence over a population’s phenotypic composition than chemical mutagenesis had in promoting genetic diversity within the population, resulting in several specific mutation patterns becoming enriched over time. Additionally, this method can be utilized as a tool for the discovery and characterization of specific bacteriophage traits for the purpose of posterior fine-mapping.

Characterization of the thermal stability of the phages that had their mutations recapitulated by genome recombination confirmed the ability to use the mutation data produced by this method as an instructional tool for the genetic engineering of bacteriophages. The application of the CAVE protocol under a given selection criteria does in fact provide information for how the genome of a given phage can be precisely modified in order to induce desired changes in fitness or functionality. The potential to introduce multiple types of functional modifications is suggested by the mutation sites of a given selection criterion being concentrated at specific genes, as seen in our genomic analyses, suggesting a degree of functional orthogonality between gene groups. A powerful consequence of this stands out: if we can reproduce the effects of directed evolution by reintroducing the observed mutations into a wild type phage genome, then the patterns in mutation data that arise from parallel applications using different selection criteria can be combined in order to engineer a phage with multiple types of phenotypic improvements.

As demonstrated by the results of our analyses, the CAVE platform provides a broadly applicable method for developing antimicrobial tools on an efficient time scale. We expect that a variety of selection criteria can be utilized within this framework due to its mechanistic generality, highlighting the versatility of this platform for exploring many pathways upon the evolutionary landscapes of viruses. Furthermore, this technique’s ability to modify species from a range of different phage-host systems provides a variety of potential use-cases within different therapeutic and industrial contexts.

## Methods

### Materials

The host cells used in this work were *E. coli* of the Rosetta strain (for amplification of T3 and T7 phages), and *Salmonella enterica* (for amplification of NBSal001 and NBSal002). Cells were grown in Luria broth. Bacteriophage lysates were prepared by growing host cells to a concentration of $5\times 10^8$ CFU/mL and initiating infection through the addition of 50 $\upmu$L phage stock of variable concentration to the host culture, and allowing lysis to proceed to completion. The chemical mutagen used in this study was ethyl methanesulfonate (EMS).

### Mutagenesis

EMS was diluted in Dulbeccos phosphate-buffered saline (DPBS) to yield desired stock concentration (1 mM used in initial T3 mutagenesis series, 180 mM used in subsequent T3 and T7 series). These two concentrations were chosen after experimental determination of the mutagen exposure limit that phages could maintain lytic activity under; an EMS concentration increase 180-fold relative to that used in initial experiments was found to be an optimal concentration for conferring rich mutation induction, with no measurable disruption of lytic activity. These concentrations serve to illustrate the lower and upper bounds for controlled mutagenesis trajectories within the framework of the CAVE pipeline. 100 $\upmu$L phage stock ($10^9$–$10^{10}$ PFU/mM) was mixed into wells of a Tecan microplate reader with 50 $\upmu$L diluted EMS. The phages were then incubated with the mutagen at 37 $^{\circ }$C in plate reader for 1 h, with rapid shaking. 20 $\upmu$L of mutagenized phage was then mixed into another microplate well with a 180 $\upmu$L solution of host bacteria (0.5 OD600), then allowed to reach complete lysis. 100 $\upmu$L lysate was then removed, and heated in a thermocycler at a temperature of pronounced denaturation. 100 $\upmu$L of heat-selected phages were then added to another microplate well with 100 $\upmu$L fresh bacteria host (0.5 OD600), and lysis was allowed to complete. This process of mutagenesis and selection was repeated up to 30 times, with a gradual increase of the selection temperature from 50 to 68 $^{\circ }$C.

### Generation of single phage mutants

The wild-type T3 phage genome was used as a template to PCR-amplify 5 DNA fragments covering the whole phage genome with  40 bp overlapping ends. The primers used to amplify the DNA fragments can be found in Supplementary Table S2. These fragments were gel-purified and Gibson-assembled into a circular genome. The circular genome was then electroporated into DH10B *E. coli* electrocompetent cells. After recovering at 37 $^{\circ }$C, the bacterial cells were plated into soft agar and incubated overnight. Observation of plaques confirmed successful rebooting of the assembled phage genome. For the phage variants, different sets of primers were used to introduce the specific mutations in the respective DNA fragments; the assembly and phage rebooting process was carried out without any further modification. The primers used to generate the specific mutations can be found on Supplementary Table S3. To corroborate the introduction of the desired mutations, Sanger sequencing was utilized, using the primers listed in Supplementary Table S4.

### Quantification of phage titer using spot-tests

Starting with a phage stock solution, serial dilutions were performed using LB media. Host bacteria was mixed into each diluted phage solution, and gently applied to the surface of an agarose plate in duplicate in order to form small drops, which were allowed to air-dry. Agarose plates were then placed in an incubator at 37 $^{\circ }$C for 5 h. After bacterial growth and plaque formation became visibly distinguishable, the number of plaque forming units in each dilution group were counted. Using Microsoft Excel’s LINEST function, a linear regression was performed on the dilution group’s colony forming unit count over the dilution fraction from stock; in this regression, the y-intercept was held equal to zero, and the slope value provided the concentration of the stock solution in units of PFU/mL (Supplementary Figure S11).

### High-temperature stability analysis

Two or more PCR tubes were filled with high titer stock of bacteriophages suspended in DPBS. The tubes for the thermal incubation condition were placed in a Bio Rad MyCycler thermal cycler system, and were kept at a constant temperature between 60 and 70 $^{\circ }$C for 1 h, control group tubes were kept at 25 $^{\circ }$C for 1 h. The titer of each group was then quantified using the aforementioned spot-test protocol, and the surviving fraction of active bacteriophages was found by dividing the titer of thermal selection groups by the titer of the low temperature control group. For initial thermal stability assays (Fig. 2a), an initial titer of $1 \times 10^{10}$ PFU/mL was used. In time-dependent degradation assays (Fig. 2b), an initial titer of $1 \times 10^{8}$ PFU/mL was used.

### Low-pH stability analysis

Wild type and 30th generation mutant T3 phages were subjected to a 30 min incubation in DPBS, which had been adjusted to a pH 7.0 control condition, as well as denaturant conditions of pH 3.5 and 3.7. Following the incubation period, plating experiments were done to quantify the number of viable phage particles.

### Growth and infection curves

All growth and infection curves were measured using a Tecan microplate reader. All experiments used LB broth as the growth medium, and optical density measurements were collected at five min intervals.

### Calculation of thermodynamic and kinetic parameters

Initial concentrations for all groups were diluted in DPBS to a titer of approximately $1 \times 10^8$ PFU/mL. Phage solution was added to PCR-tubes and placed in a thermocycler set to 60, 62, and 64 $^{\circ }$C. Over the course of 1 h, samples were removed from the thermocycler every 10 min, and the titer at each time point was quantified using serial-dilution spot tests.

Following methods illustrated in previous literature^22^, these data were mapped to their corresponding timepoints by performing a nonlinear least squares calculation with a logarithmically transformed version of our kinetic model of thermal degradation (Eq. 1). The regression was performed using Python and the scientific analysis toolkit SciPy^26^, employing a Trust Region Reflective Algorithm^27^ to obtain the kinetic and thermodynamic parameters (*n*, $k_{ref}$,and $E_a$) . The reference temperature used in the regression calculation was 62 $^{\circ }$C. The acquired parameters were used to provide theoretical denaturation curves (Fig. 1b). Kinetic parameter values were also used to calculate theoretical half life values (Fig. 1c), using Eq. (2). For a known temperature and kinetic parameters, uncertainty in the calculated half life value can be propagated as a function of uncertainty in the measurement of initial concentration,

1 $$\begin{aligned} \sigma _{t_{1/2}} = \sqrt{\left( \frac{\partial t_{1/2}}{\partial N_{0}}\sigma _{N_{0}} \right) ^{2}} \end{aligned}$$

where the partial derivative of half-life with respect to initial titer is as follows:

2 $$\begin{aligned} \frac{\partial t_{1/2}}{\partial N_{0}} = \frac{(1-2^{n-1})e^{\frac{E_{a}}{R}\left( \frac{1}{T}-\frac{1}{T_{ref}}\right) }}{k_{ref}N_{n}^{0}} \end{aligned}$$

### Bacteriophage genome purification

An initial volume of 10 mL high-titer phage lysate was produced by infecting host bacteria with a phage of interest. Residual bacterial cells were removed via centrifugation at 4,000*g* for 10 min. Phage-solutions were passed through a 1 $\upmu$m syringe-filter for further purification. The solution was then mixed with 1 equivalent PEG/NaCl per 2 equivalents lysate. After mixing, the solution was left in a refrigerator overnight to precipitate.

Following precipitation, the solution was centrifuged at 10,000 rpm for 1 h, and supernatant was carefully removed in order to preserve the resulting phage pellet. The phage pellet was resuspended in 500 $\upmu$L $\hbox {MgCl}_2$. 2.5 $\upmu$L RNaseA and DNaseI were added to the 500 $\upmu$L suspension, and incubated at 37 $^{\circ }$C for 1 h. 2.5 $\upmu$L Proteinase-K, 25 $\upmu$L 10% SDS and 20 $\upmu$L 0.5M EDTA were then added, gently mixed, and left to incubate at 60 $^{\circ }$C for an additional hour. An equal volume of phenolchloroform was then added to the enzyme-treated solution. Following gentle mixing via inversion, the solution was centrifuged at 3,000*g* for 5 min. Three iterations of aqueous-layer purification with phenolchloroform were performed. For the remaining aqueous-layer volume, 1/10th volumetric equivalent of 5M NaCl, and 2.5 times the equivalent of pure ethanol were added, gently mixed, and then left at $-\,20\,^{\circ }$C overnight.

The solution was then centrifuged at 3,000*g* for 1 h to pellet the precipitated DNA. Supernatant was removed, and the DNA pellets were resuspended in 70% ethanol. Centrifugation and ethanol-resuspension steps were repeated twice to further purify the DNA. After the final centrifugation step, ethanol was removed, and the DNA pellet was left to air-dry for 30 min. The dry DNA pellet was resuspended in Buffer EB (Qiagen), and submitted for sequencing.

### Processing and analysis of next-generation sequencing data

Genomes submitted for sequencing corresponded to the wild-type or mutant samples from generations 0 (wild type), 10, 20, and 30 of the initial T3 mutagenesis series, and generations 0 (wild type), 5, 10, and 15 of the T3/T7 parallel evolution series (12 samples total). The purified genomes were sequenced by QuickBiology Inc. (HiSeq X, 5M reads total per sample, 2.5M pairs, 2 $\times$ 150 PE), and raw data for reads aligned to the wild type reference genome were provided in the form of fastq.gz files. The fastq data for the mutant genome pools were processed using the following pipeline:

1. The raw paired-end reads were trimmed using Trimmomatic-0.38)^28^, limiting the minimum length to 30, and using a sliding window of 2:30.
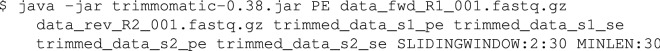
2. Paired-end reads were merged with (FLASH-1.2.11)^29^.
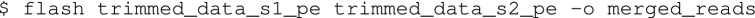
3. The reference fasta was indexed, and then used for alignment of the merged sequencing file using the Burrows–Wheeler Aligner (bwa-0.7.17)^30^, to generate a SAM file.
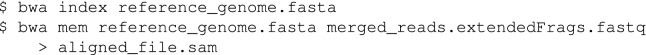
4. The SAM file was then converted to a BAM file, sorted, and indexed using (Samtools-1.9)^31^.
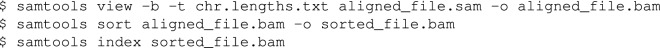
5. Variant calling for the sorted BAM file was performed using LoFreq^32^, a high-sensitivity variant calling program that is optimal for analysis of small genomes with high mutational variance.
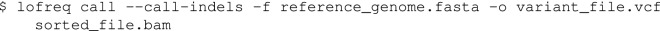

In order to analyze the the allele-frequencies produced by our NGS data-processing pipeline, Variant Call Format files were opened in Python3 using the Numpy^33^ library. Mutant allele-frequencies for each round of CAVE were normalized to a baseline by subtracting the background variant frequencies found in NGS data for wild type samples (negative controls). Gene mutation indices were calculated by summing the allele frequencies for bases within the region spanning a given gene. All figures displaying mutation frequencies across genomes were produced by plotting the normalized allele frequencies, using Matplotlib^34^.

### Structural analysis of highly mutable proteins

PDB files for the head-to-tail joining protein, tail-tubular protein A, and tail-tubular protein B were downloaded from the the structure of PDB ID: 6R21, which was published by Cuervo et al.^24^. Visualization and coloring of the structures was done at the Molecular Graphics and Computation Facility at the University of California, Berkeley, which is supported by the National Institute of Health, under award number S10OD023532.

## Supplementary information

Supplementary information.

## Data Availability

All experimental data are included in this article and its supplementary materials. All NGS data associated with this study have been deposited in the NCBI Sequence Read Archive (SRA), and can be accessed from the SRA Run Selector. Metadata for the associated analyses have been reported on NCBI, with BioProject Accession PRJNA631837.
