# Supplementary material for: Optimizing bacteriophage engineering through an accelerated evolution platform
Source: Sci Rep. 2020 Aug 19;10:13981. doi: 10.1038/s41598-020-70841-1 (PMC7438504; doi:10.1038/s41598-020-70841-1)
Supplement: Supplementary file 1 — Supplementary information. [file 41598_2020_70841_MOESM1_ESM.pdf]

Optimizing bacteriophage engineering through an accelerated evolution platform

Andrew H. Favor[1,2], Carlos D. Llanos[1], Matthew D. Youngblut[1], and Jorge A. Bardales[1]

[1]Nextbiotics Inc. Oakland, CA 94609, USA. [2]Current address: department of Materials Science and Engineering, University of California, Berkeley, CA 94720, USA.

Supplemental Discussion

Kinetic model of thermal denaturation

In considering the dynamics of thermal denaturation, it is important to note the nonstandard kinetic order of such processes, as described in previous literature [1–5]. The n-th order integrated rate equation provides a description of the decrease in concentration of active phage over time, as a function of starting concentration and temperature, as per the Arrhenius relation [6].

$$N(t) = N_0 \left(1 + k_{ref}(n-1)tN_0^{n-1}e^{\frac{E_a}{R}\left(\frac{1}{T_{ref}}-\frac{1}{T}\right)}\right)^{\frac{1}{(1-n)}} \tag{1}$$

After finding the values of n, k_{ref}, and E_a using a non-linear least squares optimization (see methods), these values can be used to calculate the half life of a phage variant for a given temperature as a function of initial concentration, using the following formula:

$$t_{1/2}(N_0) = \frac{(2^{n-1}-1)e^{\frac{E_a}{R}\left(\frac{1}{T}-\frac{1}{T_{ref}}\right)}}{k_{ref}(n-1)N_0^{n-1}} \tag{2}$$

Noting the dependence of degradation rate upon concentration at the initial time of thermal treatment, we may take note of the difference in the fraction of remaining wild-type phage after one hour at $60°C$ between figures **(1a)** and **(1c)** , by considering the difference in the initial concentrations used in these two experiments (1a initial titer: 10^{10} PFU/mL, 1c initial titer: 10^8 PFU/mL). While the theoretical prediction of half life values for reaction orders of $n > 1$ increases as initial concentration decreases, our experiments found that survival rates and associated half lives were higher in experiments with higher initial concentration. We hypothesize that this is due to the protein-rich environments of high titer solutions having more molecular resources to distribute the input heat throughout, leading to a diminished ability to reach the activation energy for molecular denaturation among each individual protein affected.

Degradation at different temperature ranges

Our assays for quantifying degradation rates monitored both short term degradation at high temperatures and long term degradation at low temperatures. Acknowledging the multiplicity of modes for energy input to be taken by bacteriophage molecular structures, and the innumerous denaturation pathways that can occur, a single energetic mechanism as described by our theoretical Arrhenius model is unlikely to adequately describe all classes of thermal denaturation. Likewise, the parametric fitting determined at low temperatures and high temperatures were inconsistent in value, suggesting that different mechanisms are responsible for the degradation process under vastly different thermal conditions.

Changes to pH sensitivity

Despite not applying a low-pH tolerance selection criteria in our protocol, the mutations that arose to confer heat resistance appeared to also improve survivability in acidic conditions. This effect can be rationalized as being related to changes to general structural stability, rather than specific changes in temperature sensitivity. In consideration of the limitations of bacteriophage therapy, which was a primary motivator for the development of this technique, these observations suggest that application of the CAVE protocol with high-temperature selection criteria can produce variant phages with tolerance to acidic conditions. The functional effects of such changes suggest that this method holds great promise for improving the efficacy of phages as orally administered therapeutic

agents by promoting improvements in survivability under the acidic conditions of animal digestive systems, and thereby allow more active phages to reach their target pathogens.

Changes in plaque size

Previous analyses of the phenotypic traits that contribute to variation in plaque morphology have described that larger amorphous plaques are associated with lower binding efficiency of phages to their hosts [7–9]. This rationale provides a logical explanation in light of the physical principles that govern binding and diffusivity of interacting components within a system; if phages bind less efficiently to their hosts, then they will have more free diffusion through an aqueous medium, ultimately leading to larger regions of access and less uniform geometric distributions within their infections of the regions of bacterial occupancy. Analysis of mutation enrichment led us to note that the change in plaque size coincided with the occurrence of a mutation (33152[C>T]) in the tail fiber protein (T3p48), supporting the hypothesis that this morphological change is related to host adhesion. While an increase in adhesion propensity leads to noticeably decreased spread of phages when dried upon the surface of an agarose plate, we expect that this would not limit phage diffusion significantly in the context of liquid media (such as the internal environmental targets relevant to phage therapy), and would therefore not present limitations in phages' ability to reach the target hosts within their general vicinity. This is supported by our assays of infection dynamics in liquid media **(Supplemental figure 2)**, where no difference in the rate at which infection spread between wild type and mutant phages.

Supplemental Results

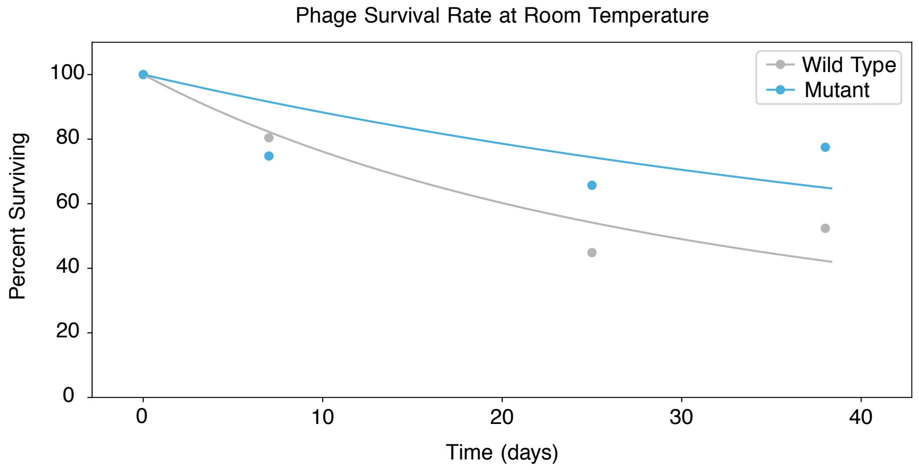

Supplementary Figure 1:

Phage degradation at room temperature.

Wild-type and mutant bacteriophages were kept at room temperature ($25°C$) for 38 days in Dulbecco's phosphate-buffered saline, with a starting concentration of 1×10^8 PFU/mL. In this time, the mutant viruses which had been selected for improved thermal stability maintained higher levels of activity, indicating an improved shelf life relative to the wild-type. Relative concentrations were calculated using plaque-counting assays and linear regressions. Theoretical curves were fit to experimental data using a fractional-order kinetic model and the Arrhenius Law.

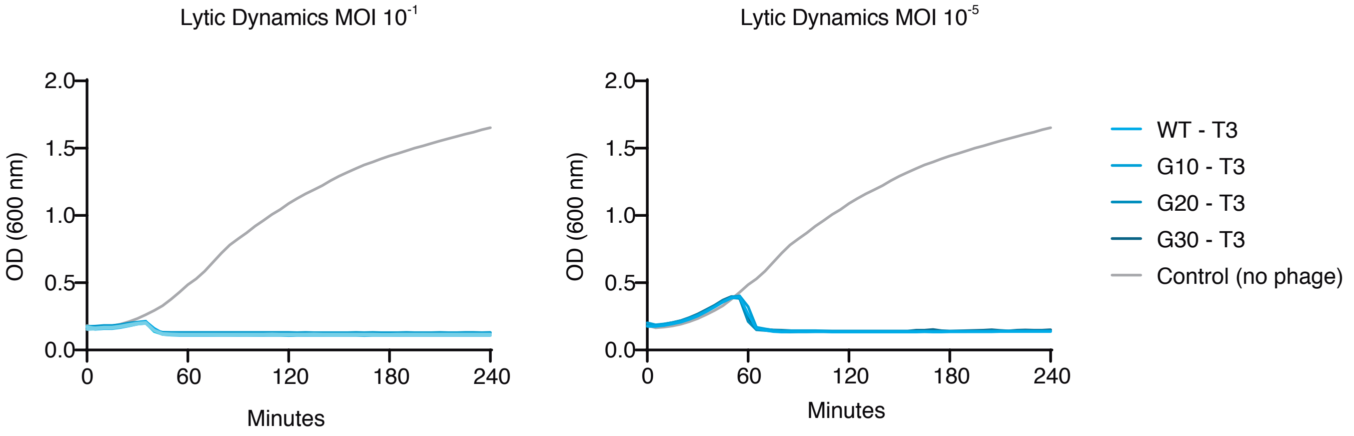

Supplementary Figure 2:

Directed evolution does not affect infection dynamics.

Infection dynamics maintain consistency between wild type phages and mutants having undergone 30 rounds of CAVE protocol. Growth curves represent lysis of host strain (Rosetta) cells, by the wild type T3 phage, and mutants from 10, 20, and 30 rounds of directed evolution. Infections were performed at multiplicities of infection of 10^{-1} (left) and 10^{-5} (right).

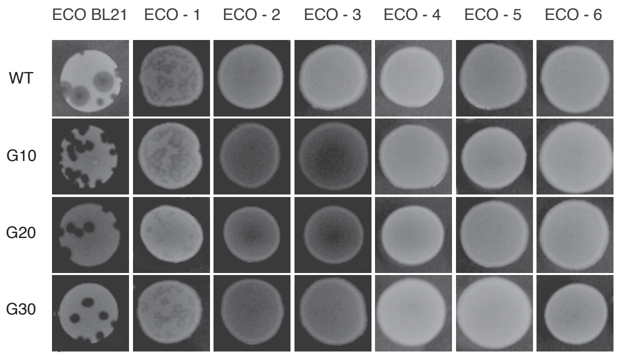

Supplementary Figure 3:

Directed evolution does not affect host range.

Host ranges maintain consistency between wild type phages and mutants having undergone 10, 20, and 30 rounds of CAVE protocol. Columns represent wild type compared to mutant phages from specified number of mutagenic protocol iterations. Rows represent the host strain that lytic activity was assayed for, through coincubation.

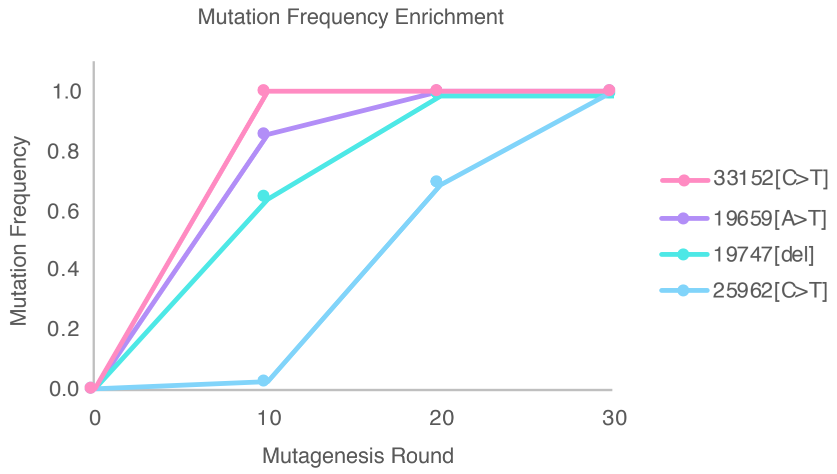

Supplementary Figure 4:

Mutation enrichment over 30 rounds of CAVE application.

Enrichent of specific mutations within the mutant pool over the course of 30 rounds of CAVE application (initial T3 mutagenesis series). Primary conserved mutations appear within the first 10 rounds of mutagenesis, and reach enrichment saturation within the entire mutant pool shortly after.

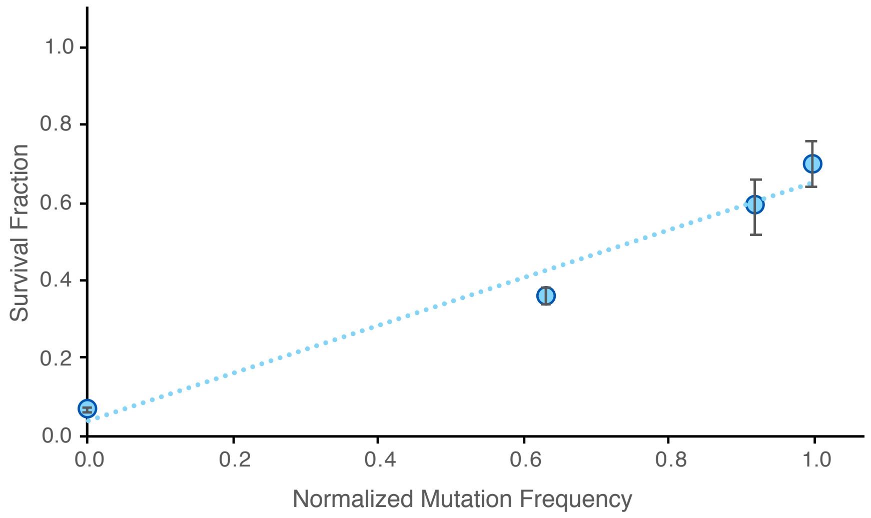

Supplementary Figure 5:

Survival vs. Mutation Frequency.

Survival fraction of T3 phages after 1 hour incubation at $60°C$ as a function of normalized mutation frequency ($R^2 = 0.968$). Normalized mutation frequency calculated as the sum all mutation frequencies across a genome at a given round of CAVE applications, divided by the total net mutation frequency achieved at the final round of evolution. A linear correlation indicates the change in thermal tolerance being induced as a function of mutation enrichment.

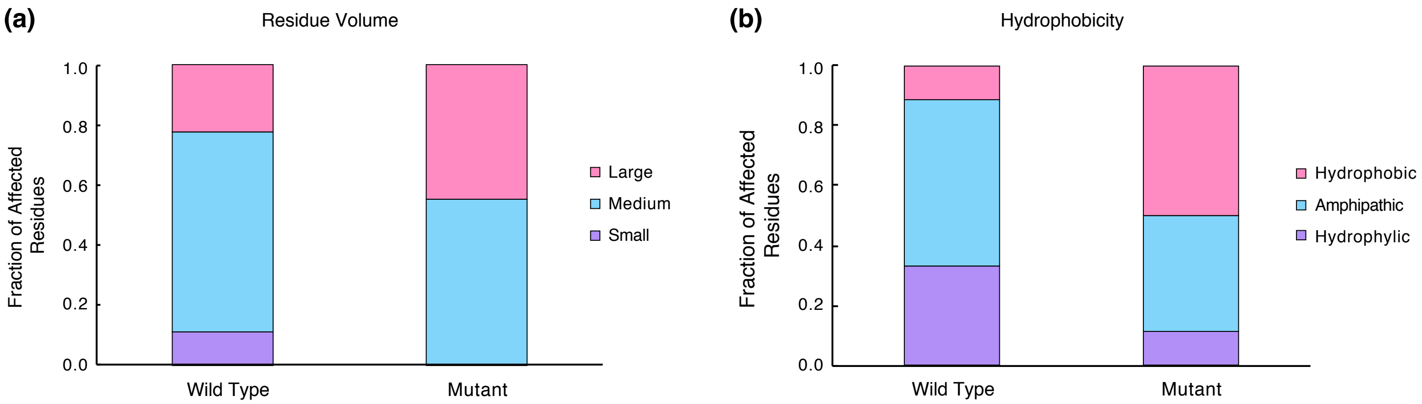

Supplementary Figure 6:

Characteristics of Substituted Amino Acids

Physical characteristic groupings of amino acids at mutated residues; comparison between wild type and mutant genome data. Amino acid data compiled from both first and second T3 mutagenesis series, as well as the T7 mutagenesis series. Amino acids were grouped based on tabulated values of volume [10] and hydrophobicity [11].

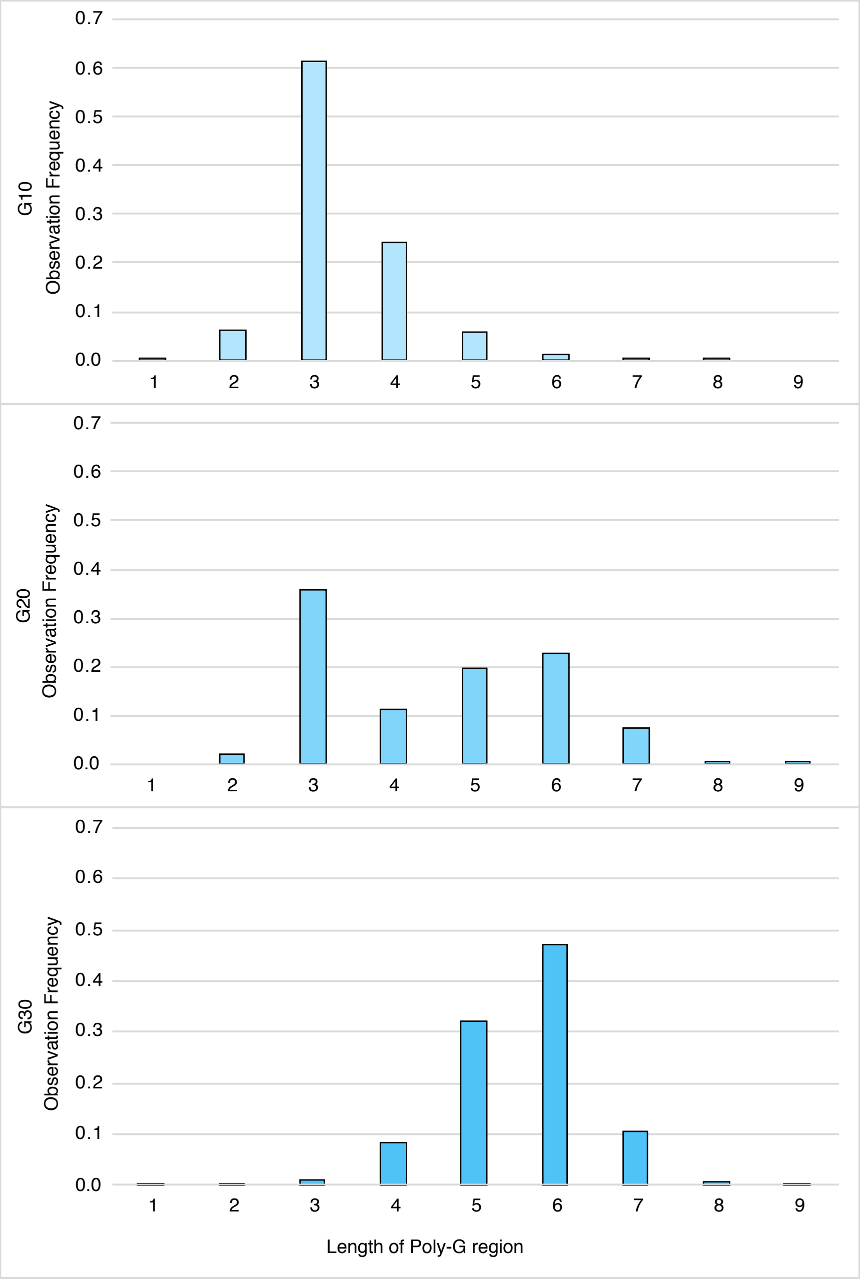

Supplementary Figure 7:

Distribution of poly-G region length over the course of directed evolution .

The observation frequency of a variable-length region of guanines changed over the course of directed evolution to be elongated. Mutant genome pools of the T3 bacteriophage were collected after 10, 20, and 30 rounds of directed evolution.

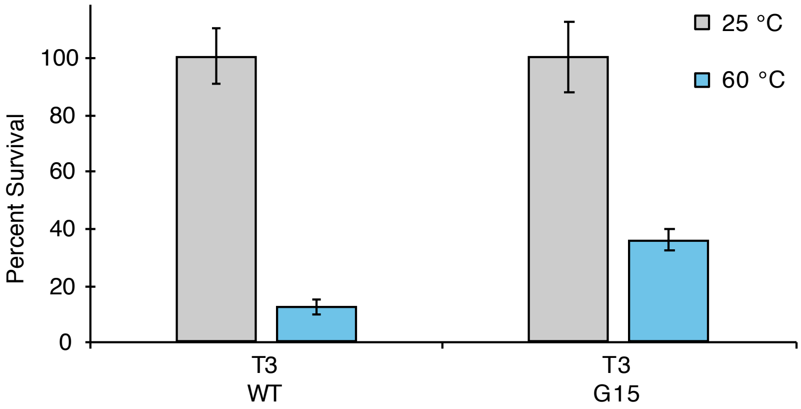

Supplementary Figure 8:

Improvement in T3 thermal stability, second mutagenesis series.

Comparison of wild type vs. mutant T7 bacteriophage (after 15 rounds of directed evolution); percent survival following 1-hour incubation at 25 °*C* and 60 °*C*.

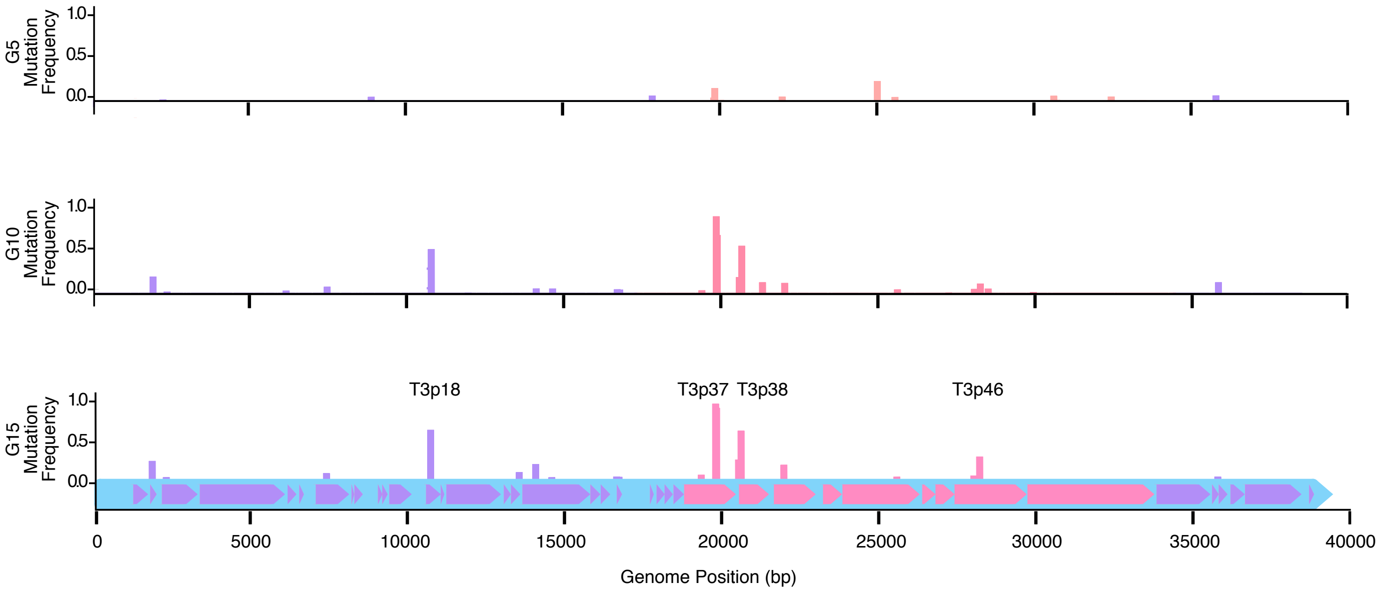

Supplementary Figure 9:

Mutations across T3 genome, second CAVE series.

Mutation frequencies observed in the T3 mutant gene pools after 5, 10, and 15 rounds of directed evolution. Mutation-frequency bars are color-coded corresponding to their functional genome location of occurrence: blue are non-coding regions, magenta are structural genes, and purple are regulatory genes.

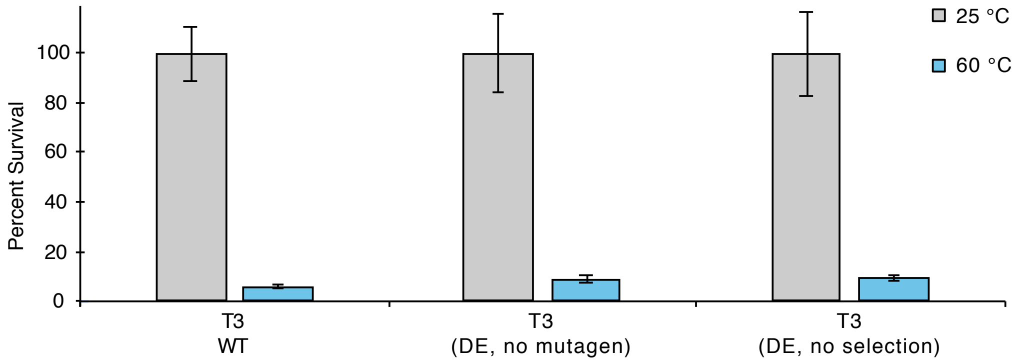

Supplementary Figure 10:

Control testing of factors involved in CAVE efficacy.

Comparison of the percent survival following 1-hour incubation at 25 °*C* and 60 °*C* for: wild type T3 bacteriophage, T3 phage subjected to 20 rounds of directed evolution in the absence of chemical mutagen, and T3 phage subjected to 20 rounds of directed evolution in the absence of thermal selection.

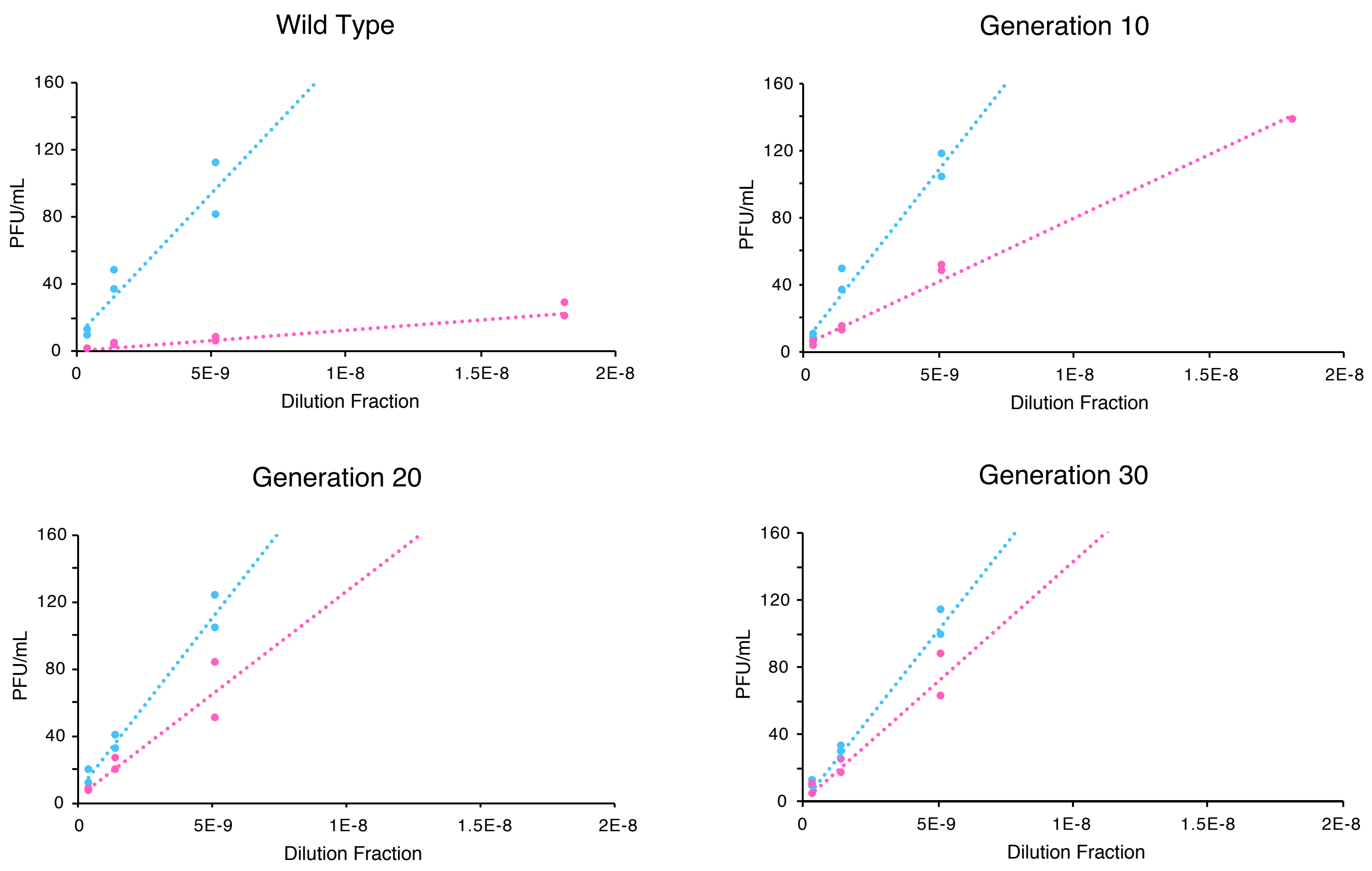

Supplementary Figure 11:

Quantification of survivability

Plaques counted vs. dilution fraction of 1 mL stock. Serial dilutions were used to quantify survival of phages following thermal incubation at 60 degrees celsius. Slopes fit via a linear regression model correspond to the phage concentration (in units of PFU/mL) in the stock solution.

Phage (CAVE Series)	Genome Position	Gene ID (Gene Name)	Mutation Frequency
T3 (series 1)	19659	T3p37 (head-to-tail joining protein)	0.998
	25962	T3p45 (internal virion B)	0.713
	33152	T3p48 (tail fiber protein)	0.197
T3 (series 2)	1723	T3p04 (gene 0.65 protein)	0.282
	10585	T3p19 (gene 3.7 protein)	0.672
	13929	T3p25 (DNA polymerase)	0.243
	19658	T3p37 (head-to-tail joining protein)	0.999
	19688	T3p37 (head-to-tail joining protein)	0.944
	20469	T3p38 (capsid assembly protein)	0.658
	21835	T3p39 (minor capsid protein)	0.234
	28066	T3p46 (internal virion protein C)	0.337
T7 (series 2)	9768	T7p17 (DNA binding protein)	0.838
	20917	T7p42 (head-to-tail joining protein)	0.930
	22828	T7p43 (capsid assembly protein)	0.720
	26118	T7p47(tail tubular protein B)	0.951
	32893	T7p51 (internal virion protein D)	0.935
	33479	T7p51 (internal virion protein D)	0.930
	38743	T7p57 (DNA maturation protein)	0.877

Supplementary Table 1: Mutations occurring within coding regions of bacteriophage genome as a result of CAVE application, initial evolution series (T3) and second evolution series (T3 and T7).

T3 Genome primers		
Primer Name	Primer Sequence	Complimentary Sequence
NbT3-P1-F2	AGTACATCTCTATGTGTCCCTTCTCATAGTTCAAGAACCCAAAGTACCCCCCCC	TCTCATAGTTCAAGAACCCAAAGTACCCCCCCC
NbT3-P1-R2	ACGAAGAAAGGCCCACCCGTGAAGGT	ACGAAGAAAGGCCCACCCGTGAAGGT
NbT3-P2-F2	ACAACCCTCAAGAGAAAATGTAACCAACTCACTGGCTC	ACAACCCTCAAGAGAAAATGTAACCAACTCACTGGCTC
NbT3-P2-R2	GGACCTTTATGTTGTTCCCACACCCATCGATGAAACATAA	GGACCTTTATGTTGTTCCCACACCCATCGATGAAACATAA
NbT3-P3-F1	*TAAAGGTGAGAAGAAACACTTTATGTTTCATCGATGGGTGTGG*	*TTATGTTTCATCGATGGGTGTGG*
NbT3-P3-R1	*GTTATAGTTGCCGTAGTCTAGCTCGTACTCGAAGCACGCA*	*GCTCGTACTCGAAGCACGCA*
NbT3-P4-F1	*TGCGTGCTTCGAGTACGAGCTAGACTACGGCAACTATAACATG*	*TAGACTACGGCAACTATAACATG*
NbT3-P4-R1	*CGCTCTACGGTAGATAGTGTAGCGAGTTTCTTCACGGGAC*	*AGCGAGTTTCTTCACGGGAC*
NbT3-P5-F1	*GTCCCGTGAAGAAACTCGCTACACTATCTACCGTAGAGCG*	*ACACTATCTACCGTAGAGCG*
NbT3-P5-R1	*TGGGTTCTTGAACTATGAGACTCTTTAAGTCTCCAATAGGCA*	*CTCTTTAAGTCTCCAATAGGCA*

Supplementary Table 2: Primers used to amplify regions of the wild type T3 bacteriophage genome.

Mutant Primers	
Primer Name	Primer Sequence
NbT3-P3-p37mut-F1	TACCTCAAGTCCAGAAGCCTTGCAAGGTGCTGCTGCACAG
NbT3-P3-p37mut-R1	TGTGCAGCAGCACCTTGCAAGGCTTCTGGACTTGAGGTAG
NbT3-mut44 -F	GGAGACACTAATAGATACGAaGGGGGGGGGTTAAAGCATTATG
NbT3-mut44 -R	AATGCTTTAACCCCCCCCCtTCGTATCTATTAGTGTCTCCCTTTAG
NbT3-P4-p45mut-F1	ATGGCAGCGATTCCTATTGTTATGGCGGGTGCCCAAGCTC
NbT3-P4-p45mut-R1	AGCTTGGGCACCCGCCATAACAATAGGAATCGCTGCCATC
NbT3-P5-p48mut-F1	TCGCTCAGATTCAAACGATTCACGTAGCGGAAGAGGCCCG
NbT3-P5-p48mut-R1	GGGCCTCTTCCGCTACGTGAATCGTTTGAATCTGAGCGAC

Supplementary Table 3: Primers used to generate specific mutations in a T3 bacteriophage genome.

Mutant "check" primers for Mutants 37, 38, 44, 45, 48-1. 48-2 (junction primers)	
Primers Name	Prime Sequence
NbT3-P3-p37/38mutCheck-F1	TTGGTCGTGGTCAAGACCTC
NbT3-P3-p37/38mutCheck-R1	CATCGCCATCACGGGCAGCA
NbT3-p44mutCheck-F	GTGCACGATTGGGGTCTGAC
NbT3--44mutCheck-R	GCTTAGCATCGCCACAGTGT
NbT3-P4-p45mutCheck-F1	CGAGTATGGTTCCTTACCTCT
NbT3-P4-p45mutCheck-R1	CGCTTGATGCGCTTCATGGAT
NbT3-P5-p48mutCheck-F1	CAATCGTGATTTTAATATCCCGTT
NbT3-P5-p48mutCheck-R1	TTTGTGTTCGTAGCGTTAGT
*mutant 37check primers was used for both mutant37 and mutant38	

Supplementary Table 4: Primers used to validate the presence of specific mutations within a mutant T3 bacteriophage genome using Sanger sequencing.

References

[1] M Koka and EM Mikolajcik. “Kinetics of thermal destruction of bacteriophages active against Streptococcus cremoris”. In: *Journal of Dairy Science* 50.7 (1967), pp. 1025–1031.

[2] Carmen Madera, Cristina Monjardın, and Juan E Suárez. “Milk contamination and resistance to processing conditions determine the fate of Lactococcus lactis bacteriophages in dairies”. In: *Appl. Environ. Microbiol.* 70.12 (2004), pp. 7365–7371.

[3] Andrea Quiberoni, Daniela M Guglielmotti, and Jorge A Reinheimer. “Inactivation of Lactobacillus delbrueckii bacteriophages by heat and biocides”. In: *International journal of food microbiology* 84.1 (2003), pp. 51–62.

[4] Roman Buckow et al. “Predictive model for inactivation of feline calicivirus, a norovirus surrogate, by heat and high hydrostatic pressure”. In: *Appl. Environ. Microbiol.* 74.4 (2008), pp. 1030–1038.

[5] M Müller-Merbach, T Rauscher, and J Hinrichs. “Inactivation of bacteriophages by thermal and high-pressure treatment”. In: *International dairy journal* 15.6-9 (2005), pp. 777–784.

[6] Svante Arrhenius. “Über die Reaktionsgeschwindigkeit bei der Inversion von Rohrzucker durch Säuren”. In: *Zeitschrift für physikalische Chemie* 4.1 (1889), pp. 226–248.

[7] Lingchong You and John Yin. “Amplification and spread of viruses in a growing plaque”. In: *Journal of theoretical biology* 200.4 (1999), pp. 365–373.

[8] John Yin and JS McCaskill. “Replication of viruses in a growing plaque: a reaction-diffusion model”. In: *Biophysical journal* 61.6 (1992), pp. 1540–1549.

[9] Stephen T Abedon and Rachel R Culler. “Bacteriophage evolution given spatial constraint”. In: *Journal of Theoretical Biology* 248.1 (2007), pp. 111–119.

[10] WR Krigbaum and Akira Komoriya. “Local interactions as a structure determinant for protein molecules: II.” In: *Biochimica et biophysica acta* 576.1 (1979), pp. 204–248.

[11] JM Zimmerman, Naomi Eliezer, and R Simha. “The characterization of amino acid sequences in proteins by statistical methods”. In: *Journal of theoretical biology* 21.2 (1968), pp. 170–201.
